# The Relationship Between Barriers to Physical Activity and Depressive Symptoms in Community-Dwelling Women

**DOI:** 10.1089/whr.2023.0034

**Published:** 2024-03-13

**Authors:** Caroline A. Figueroa, Adrian Aguilera, Thomas J. Hoffmann, Yoshimi Fukuoka

**Affiliations:** ^1^ Department Engineering Systems and Services, Faculty of Technology, Policy, and Management, Delft University of Technology, Delft, The Netherlands.; ^2^ School of Social Welfare, University of California, Berkeley, California, USA.; ^3^ Department of Epidemiology and Biostatistics, and Office of Research, School of Nursing, University of California, San Francisco, San Francisco, California, USA.; ^4^ Department of Psychiatry and Behavioral Sciences, Zuckerberg San Francisco General Hospital, University of California, San Francisco, San Francisco, California, USA.; ^5^ Department of Physiological Nursing, School of Nursing, University of California, San Francisco, San Francisco, California, USA.

**Keywords:** physical activity, depressive symptoms, women's health, preventive medicine, exercise

## Abstract

**Background:**

Women are less physically active, report greater perceived barriers for exercise, and show higher levels of depressive symptoms. This contributes to high global disability. The relationship between perceived barriers for physical activity and depressive symptoms in women remains largely unexplored. The aims of this cross-sectional analysis were to examine the association between physical activity barriers and depressive symptoms, and identify types of barriers in physically inactive community-dwelling women.

**Methods:**

Three hundred eighteen physically inactive women aged 25–65 years completed the Barriers to Being Active Quiz (BBAQ) developed by the Centers for Disease Control and Prevention, and the Center for Epidemiological Studies Depression Scale at the baseline visit of the mobile phone-based physical activity education trial. The BBAQ consists of six subscales (lack of time, social influence, lack of energy, lack of willpower, fear of injury, lack of skill, and lack of resources). We used multivariate regression analyses, correcting for sociodemographics.

**Results:**

Higher physical activity barriers were associated with greater depressive symptoms scores (linear effect, estimate = 0.75, 95% confidence interval [CI]: 0.39–1.12, *p* < 0.001). This effect appeared to taper off for the higher barrier scores (quadratic effect, estimate: −0.02, 95% CI: −0.03 to −0.01, *p* = 0.002). Exploratory analyses indicated that these associations were most driven by the social influence (*p* = 0.027) and lack of energy subscales (*p* = 0.017).

**Conclusions:**

Higher depression scores were associated with higher physical activity barriers. Social influence and lack of energy were particularly important barriers. Addressing these barriers may improve the efficacy of physical activity interventions in women with higher depressive symptoms. Future research should assess this in a randomized controlled trial.

**Trial Registration ClinicalTrials.gov#:**

NCTO1280812 registered January 21, 2011.

## Introduction

Despite the physical and mental health benefits of regular physical activity, women in every age group are less likely to meet the recommended levels of physical activity than men.^1–3^ For instance, according to both self-report and accelerometry measures, moderate and vigorous physical activity levels are higher in men than women.^1^ This may be, in part, because women face greater barriers to physical activity than men.^4,5^. Most common physical activity barriers in women include lack of motivation,^3^ time, and energy.^6,7^ Other reported barriers are the failure to see themselves as athletes^4^ and health problems.^8,9^ To address low physical activity in women, more knowledge on these barriers and how to overcome them is necessary.

Women also have a risk up to twofold compared to men of developing depressive symptoms.^10,11^ Evidence from longitudinal studies suggests that physical inactivity and depressive symptoms have a bidirectional relationship.^12^ Increasing aerobic exercise of any intensity can reduce mild to moderate depressive symptoms in women^13^ In turn, both having a depression diagnosis,^14^ and having mild depressive symptoms,^15^ are associated with reduced likelihood of maintaining an exercise program in men and women. In a sample of community women, the odds of depressive symptoms were lower among women who reported more leisure-time physical activity (PA).^16^ Therefore, focusing on promoting physical activity in women can substantially benefit both women's mental (*e.g.*, reduce depressive symptoms) and physical health (*e.g.*, reduce the risk of diabetes, cardiovascular disease, and cancer).^17^

It is imperative to understand if women with higher depressive symptoms report higher barriers and which unique barriers are of particular importance. For instance, barriers to physical activity may moderate the effect of physical activity interventions.^18^ There is also evidence for a dose-response relationship between the number of barriers and meeting recommendations for physical activity among women.^19^ Yet, we lack knowledge on the relationship between physical activity barriers and depressive symptoms in women who are physically inactive. Understanding barriers to physical activity, and their relationship to depressive symptoms, will help researchers, clinicians, and other stakeholders to improve the development of physical activity interventions for women.

This article aims to address this gap by examining the relationship between physical activity barriers and depressive symptoms in community-dwelling women who are physically inactive and enrolled in a mobile physical activity intervention, the mobile phone-based physical activity education (mPED).

### The aims of this study were to

(1) Examine if depressive symptoms are associated with the total barriers to physical activity score, correcting for demographics, clinical variables, and a measure of emotional support.
(2) Assess if depressive symptoms are associated with the barrier subscale scores correcting for demographics, clinical variables, and social support in an exploratory manner.

## Methods

### Study design and sample

In this cross-sectional analysis of 318 women in the mPED trial, we analyzed the sociodemographic, clinical, and self-reported questionnaire data collected at the screening/baseline study visit. Detailed descriptions of the study design and outcomes have been previously published.^20–24^ In short, eligibility criteria were female sex, age from 25 to 65 years, body mass index (BMI; calculated as weight in kilograms divided by height in meters squared) of 18.5–43.0, physically inactive at work and/or during leisure time based on the Stanford Brief Activity Survey, intent to be physically active, access to a home telephone or mobile phone, ability to speak and read English, no medical conditions or physical problems that required special attention in an exercise program, no current participation in other lifestyle modification programs, and no mild cognitive impairment as determined by the Mini-Cog test.

During the screening/baseline visit, sociodemographics, medical and lifestyle history, the Center for Epidemiological Studies Depression Scale (CES-D),^25^ BMI, and Barriers to Being Active Quiz (BBAQ) were assessed by trained research staff. All methods were conducted in accordance with the Declaration of Helsinki. The study was approved by the Institutional Review Board at the University of California, San Francisco (UCSF), and by the safety monitoring board members appointed by the research team. Written informed consent was obtained from all participants before any research procedures started.

### Measures

#### Depressive symptoms

The CES-D^25^ was used to assess self-reported depressive symptoms. The CES-D is a valid and reliable instrument that is widely used to assess depressive symptoms in a research context. The CES-D ranges from 0 to 60, with a cutoff score of 16 indicating risk for clinical depression. Higher scores indicate greater depressive symptoms.

#### Physical activity barriers scale

Barriers to Being Active Quiz developed by the Centers for Disease Control and Prevention (CDC)^26^ is a 21-item measure assessing the following barriers to physical activity: (1) lack of time, (2) social influence, (3) lack of energy, (4) lack of willpower, (5) fear of injury, (6) lack of skill, and (7) lack of resources (*e.g.*, recreational facilities, exercise equipment). Each domain contains three items, with a total score range of 0–63. Respondents rate the degree of activity interference on a 4-point scale, ranging from 0 = “very unlikely” to 3 = “very likely.” Higher scores indicate more significant barriers to physical activity.

#### Other measures

The participants filled out the sociodemographic and medical history questionnaires immediately after obtaining the written consent form. The emotional support question, “How many people can you count on to provide you with emotional support?” was developed by the research team. To calculate BMI, weight was measured with a Tanita WB-110 digital electronic scale, and height was measured at baseline with a standard stadiometer twice to check the accuracy of measurements.

### Statistical analysis

We assessed the relationship between the total CES-D score (depressive symptoms) and the total Barriers to Being Active Quiz scale score correcting for demographics, clinical variables, and emotional support. We additionally conducted a *post hoc* analysis to examine the relationship between the total CES-D score and the BBAQ subscales.

We used descriptive statistics to summarize sample characteristics and linear and logistic regression models to examine the association between the total BBAQ barriers and subscale scores CES-D with depressive symptom scores. For subscale scores with a non-normal distribution, we computed a binary variable based on the median scores. We included self-reported age, BMI, employment (paid work, yes or no), whether participants have children at home (yes/no), whether they have driven a car in the past week (yes/no), and marital status as covariates in the model, based on previous evidence.^6,20–22^ We examined nonlinear effects of depression by including the quadratic effect of depression in the models.

A quadratic effect is captured by adding a squared term of the depressive symptom scores to the regression model. Adding this effect helps to understand the more nuanced and complex interactions between depressive symptoms and physical activity barriers by accounting for the curvature in the relationship. We used likelihood ratio (LR) tests, statistical tests used to compare the goodness-of-fit of two models, to assess the need for including these nonlinear effects. We removed influential observations using Cook's *d*, which identifies potential outliers or influential observations that might be driving the model's results (see results; we also conduct a sensitivity test when the influential observations are not removed). We checked further model assumptions by visual inspections of residual plots. Tables are presented in the supplementary analysis. Analyses were carried out in R studio V. 1.1.423.

## Results

The baseline characteristics of the 318 participants and the mean or median scores of the total barriers and the subscales are shown in Table 1. For the overall sample, based on the mean and median scores, the greatest barriers were lack of time, lack of energy, and lack of willpower. Fear of injury was the most minor common reported barrier. In the Supplementary Data (Supplementary Table 1), we also show differences in the total scale and subscales between groups with high depressive symptoms (CES-D ≥ 16) for illustrative purposes (in analyses, we used the continuous scores).

**Table 1.** Baseline Sample Characteristics (*N* = 318)

	Mean (±SD), median (IQR; not normally distributed variables) or ***n*** (%)
Age^a^	
Median	54.0 (50/60/60)
Ethnicity	
Native Hawaiian/Pacific Islander	1 (0.3%)
Black/African-American	26 (8.2%)
Hispanic/Latino	20 (6.3%)
Asian	65 (20.4%)
White (non-Hispanic)	179 (56.3%)
More than 1 race	27 (8.5%)
Education	
Completed high school and some college	77 (24.2%)
Completed college	128 (40.3%)
Completed graduate school	113 (35.5%)
Annual household income	
Under $40,000	62 (19.5%)
$40,001–75,000	77 (24.2%)
Over $75,000	153 (48.1%)
Don't know or declined to state	26 (8.2%)
Marital status^b^	
Never married	99 (31.1%)
Currently married/cohabitating	158 (49.7%)
Divorced/widowed	61 (19.2%)
Employment	
No, full or part time job no shift work	160 (50.3%)
Yes full or part time job with shift work	71 (22.3%)
No paid employment	87 (27.4%)
Antidepressant	
No	246 (77.4%)
Yes	72 (22.6%)
Emotional support	
Support from ≥3 people	257 (80.8%)
BMI (kg/m^2^)	
Mean (SD)	29.6 (6.13)
Self-reported high blood pressure (%)	
No	232 (73.0%)
Yes	80 (25.2%)
Don't know	6 (1.9%)
Self-reported high cholesterol (%)	
No	175 (55.0%)
Yes	97 (30.5%)
Don't know	46 (14.5%)
Barriers scale and subscales	
BBAQ total score	25 (17/25/31)
Lack of time	4.23 (2.68)
Social influence	3.53 (2.10)
Lack of energy	4.18 (2.64)
Willpower	7.00 (6/7/8)
Fear of injury	0.00 (0/0/2)
Lack of skills	1.00 (0/1/3)
Lack of resources	2.00 (0/2/4)

^a^ Age was divided into 10-year intervals to increase interpretability.

^b^ Being married was the reference level.

BBAQ, the Barriers to Being Active Quiz; BMI, body mass index; CES-D, Center for Epidemiological Studies Depression Scale; IQR, interquartile range; SD, standard deviation.

### The total barriers to being active quiz score

**FIG. 1.**
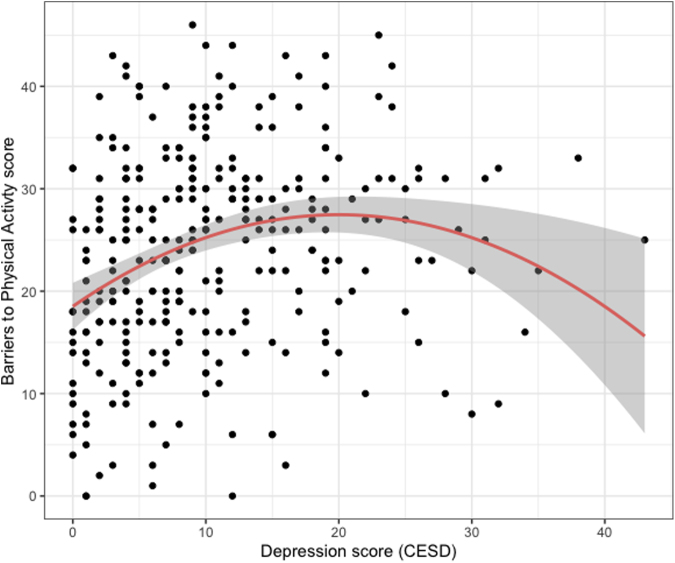
Quadratic relationship between physical activity barriers score and depression scores.

We first assessed if depressive symptoms were associated with the Total Barriers to Being Active Quiz scale score. We utilized linear regression models adjusting for self-reported age, BMI, employment (paid work, yes or no), whether participants have children at home (yes/no), whether they have driven a car in the past week (yes/no), and marital status. The total barriers scale showed a significant relationship with depressive symptoms (linear effect of depression, estimate = 0.75, 95% confidence interval [CI]: 0.39–1.12, *p* < 0.001), which tapered off for the higher barrier scores (quadratic effect of depression, estimate: −0.02, 95% CI: −0.03 to −0.01, *p* = 0.002); this relationship is best viewed in Figure 1. Other factors associated with a higher barrier scores were lower age and higher employment levels (Table 2).

**Table 2.** Multivariate Linear Regression Predicting the Barriers to Being Active Quiz Total Score

Predictors	Total barriers scale		
	Estimates	CI	***p***
(Intercept)	29.48	21.76 to 37.20	**<0.001**
Age (10-year intervals)^a^	−0.17	−0.26 to −0.08	**<0.001**
Paid full or part-time employment	2.71	0.37 to 5.04	**0.024**
Children living at home	−0.01	−2.52 to 2.50	0.992
Never married^b^	0.18	−2.17 to 2.52	0.881
Divorced/widowed^b^	−0.49	−3.24 to 2.26	0.725
Driving in past week	−0.54	−3.21 to 2.13	0.690
BMI (kg/m^2^)	−0.08	−0.24 to 0.09	0.363
CES-D score (continuous)	0.75	0.39 to 1.12	**<0.001**
Quadratic effect of depression	−0.02	−0.03 to −0.01	**0.002**

Bold means statistically significant result.

^a^ Age was divided into 10-year intervals to increase interpretability.

^b^ Being married was the reference level.

CI, confidence interval.

To ensure the robustness of these findings, we conducted several comparisons and sensitivity analysis with other models. We found that a model that included a quadratic term was a better fit than a model without this term (of note a cubic term was not significant, *p* = 0.09. In our main results, we removed four influential observations (Cook's *d*); when influential observations were retained in the model, results were similar for the linear effect (linear effect of depression, estimate = 0.38, 95% CI: 0.05–0.70, *p* = 0.023), but the quadratic effect was no longer significant (quadratic effect of depression, estimate: −0.005, 95% CI: −0.01 to 0.00, *p* = 0.23).

Next, to assess this relationship using more flexible modeling, we also examined a spline regression model. Spline regression is a nonparametric technique that divides the datasets into intervals with different fits, which may yield better results depending on the nature of the data. Results for spline regression were relatively similar but with a slower taper off at higher depression scores (Supplementary Data). LR tests showed that a model including a quadratic term was a better fit than a spline regression model.

### The barriers to being active quiz subscales

We next assessed whether depressive symptoms were associated with barriers to being physically active, again with linear regression adjusting for the same covariates. We observed a significant relationship with depression scores for the social influence subscales (linear effect of depression, estimate = 0.03, 95% CI: 0.00–0.06, *p* = 0.027, Table 3A), and the energy subscale (linear effect of depression, estimate = 0.04, 95% CI: 0.01–0.07, *p* = 0.017, Table 3B). The subscales lack of resources, lack of skill, and lack of willpower and were all marginally significant and positively associated (*p* < 0.01; Supplementary Data).

**Table 3.** Multivariate Linear Regression Predicting the Social Influence and Energy Subscale Scores

Predictors	Estimates	CI	***p***
A. Influence others			
(Intercept)	3.87	2.17 to 5.57	**<0.001**
Age (10-year intervals)^1^	−0.03	−0.05 to −0.01	**0.005**
Paid full or part-time	−0.17	−0.69 to 0.34	0.516
Employment			
Children living at home	−0.03	−0.58 to 0.53	0.928
Never married^2^	−0.26	−0.79 to 0.26	0.321
Divorced/widowed^2^	0.28	−0.33 to 0.88	0.371
Driving in past week	−0.12	−0.72 to 0.48	0.697
BMI (kg/m^2^)	0.04	0.00 to 0.08	**0.03**
CES-D scores (continuous)	0.03	0.00 to 0.06	**0.027**
B. Energy			
(Intercept)	6.13	4.13 to 8.13	**<0.001**
Age (10-year intervals)^1^	−0.06	−0.09 to −0.04	**<0.001**
Paid full or part-time	1.59	0.99 to 2.18	**<0.001**
Employment			
Children living at home	0.08	−0.57 to 0.73	0.81
Never married^2^	0.47	−0.14 to 1.08	0.135
Divorced/widowed^2^	−0.02	−0.73 to 0.69	0.955
Driving in past week	0.07	−0.62 to 0.76	0.84
BMI (kg/m^2^)	−0.01	−0.05 to 0.04	0.784
CES-D scores (continuous)	0.04	0.01 to 0.07	**0.017**

Bold means statistically significant result.

In our model comparisons and sensitivity analysis, we found that models without quadratic effects were better fits. For the social influence scale and the energy scale, we removed three influential observations based on Cook's *d*; results were similar when retaining influential observations in the model. Of note, the subscale results are in the context of an exploratory analysis and would not pass the threshold for multiple comparisons.

## Discussion

We showed that higher depressive symptom scores were associated with higher physical activity barriers in physically inactive women when adjusting for sociodemographic and clinical variables. The Social Influence and Lack of Energy, Barriers to Being Active Quiz subscales, were most associated with depressive symptoms in a *post hoc* analysis. Although we assessed these relationships in an exploratory analysis, these barriers may be the driving factors behind the differences in total subscale scores. For the sample as a whole, we identified that lack of willpower, lack of time, and lack of energy were the most frequently reported barriers to physical activity whereas injury and lack of skill were less often reported. Physical activity interventions for inactive women may be more effective when they take into account that women with higher depressive symptoms could have higher, and unique, barriers to physical activity.

### Most important barriers for physical activity

Our *post hoc* results, although they need to be confirmed in future work, suggest the need for an emphasis on social influence and boosting energy to increase the effectiveness of physical activity interventions in women with high depressive symptoms. A lack of social support has been suggested as a risk factor for physical inactivity before.^27^ Other research found that having a family member who exercises or who encourages exercise motivates engaging in healthy behaviors.^28^ Our findings also suggest that self-consciousness in social exercise-related situations (*e.g.*, appearance toward others when exercising) may be an important factor discouraging women with higher depressive symptoms from physical activity. To be effective for women with higher depressive symptoms, physical activity interventions should take these barriers into account.

Interventions could, for instance, integrate social support from family or friends, utilize peer-support or use community-based structures. Furthermore, exercise interventions can build-in graded exercise, personalized to a women's individual fitness levels to help slowly overcome feelings of fatigue.^29^ Future work should also quantify and integrate facilitators to exercise in women with high depressive symptoms. In mixed gender populations with clinical depression, facilitators included having a reason for exercising, being able to identify the psychological benefits of exercise, having positive social support and integrating cognitive behavior change strategies.^30^ It remains unclear whether these facilitators are similar in those with higher depressive symptoms.

Physical activity interventions are increasingly delivered in digital formats and *via* smartphones, using apps, text-messaging, and conversational agents. There is a growing interest in adaptive interventions, which alter their content based on the day-to-day behavior of individuals.^31^ We argue that physical activity interventions should both adapt to individuals' daily changing circumstances, and tailor their content to overcoming barriers of user subgroups.

### The quadratic effect of depression

We found that the relationship between depression and physical activity barriers was not linear, but had a more complex shape. Including a quadratic effect of depression in our regression model provided the best fit to the data. After the CES-D score of around 20, past the clinical cutoff for identifying individuals at risk for clinical depression (≥16), physical activity barriers no longer increased with higher depressive symptom scores. This suggests that beyond a certain depression level, the relationship between depression and barriers becomes less pronounced. Although we cannot be certain why this effect tapers off, there are multiple possible explanations. First, for participants with higher depressive symptom scores, physical activity may not be a priority, and therefore they are less aware of their physical activity barriers.

Another potential explanation is that Barriers to Being Active Quiz scale does not capture all relevant barriers when women reach more severe levels of depressive symptoms. For instance, previous research^32^ showed that in severe mental illness, low mood and stress are perceived as the most significant barriers for physical activity, followed by social support. In addition, in outpatients with depression, physical exertion was the most common reported PA barrier.^33^ The Barriers to Being Active Quiz scale used in this study assess lack of energy, but it does not capture whether low mood, high stress, or physical exhaustion prevent women from exercising. We recommend these questions be included in future versions.

One caveat here is that our data were sparse for very high depressive symptom scores, making these estimates less precise. Further, when we retained influential observations in the model, the quadratic effect lost significance. Future work should assess differences in barriers between women with elevated depressive symptoms and clinically diagnosed depression.

### Age, employment, and physical activity

Another interesting finding was the relationship between age and perceived physical activity barriers. In our *post hoc* analyses, we found that as age increases, physical activity barriers decrease, except for the injury and skills scales. These findings complement previous work in community samples showing that younger adults, both men and women, (25–44 years) report most physical activity barriers, and older adults (>65 years) least.^34^ In line with our findings, earlier work has also identified lack of time and energy due to family and household responsibilities among the top barriers to physical activity for women.^35^ Older women may be less impacted by these responsibilities.

Finally, full-time or part-time employment was associated with higher barriers opposed to unemployment. Past work revealed that working women perceived lack of time and energy as most frequently reported barriers to physical activity.^6^ Future interventions may want to incorporate strategies for promoting physical activity into working hours to overcome these barriers for working women.

### Strengths and limitations

We included a relatively large sample of diverse women. To our knowledge, this is one of the first studies to systematically examine the relationship between physical activity barriers using the instrument that was developed and validated by the CDC^36^ and depressive symptoms in women.

A limitation is that findings may be specific to our sample of female adults aged 25–65 years with relatively high levels of education from the San Francisco Bay Area. Our sample included fewer women with high depression scores, that is, CESD >20, therefore, the estimate of the relationship may be less reliable after these higher scores and are not applicable to women diagnosed with clinical depression. In addition, our analyses are cross-sectional, thus do not allow us to understand the causal relationship between perceived barriers and depression. Another limitation may be selection bias. Since we included women who signed up to participate in a physical activity intervention, the study participants might be more motivated to engage in physical activity. Further, women with higher depressive symptoms are less likely to participate in the study. This may, in part, explain the tapering off effects we observe for high depression scores.

Finally, the barriers scale, although designed to measure the most common physical activity barriers, contains items that overlap with aspects of depressive symptoms, such as a lack of energy. Although we corrected for potential confounding factors, it is possible that our findings reflect not only the relationship between depressive symptoms and perceived barriers, but also how depression may affect individuals' responses to the barriers scale items themselves. Future research could use alternative measures of physical activity barriers, and examine the causal relationship between barriers and depression in (quasi)-experimental designs.

## Conclusion

In community-dwelling women who enrolled in the mPED trial, higher depressive symptom scores were associated with higher physical activity barriers when correcting known confounding factors. In an exploratory *post hoc* analysis, we identified social influence and lack of energy as particularly important physical activity barriers. Addressing these barriers may improve an efficacy of physical activity interventions in women with high depressive symptoms. Further research, such as an randomized controlled trial, should confirm our findings.

## Supplementary Material

Supplemental data

## Abbreviations Used

BBAQ: the Barriers to Being Active Quiz
BMI: body mass index
CDC: Centers for Disease Control and Prevention
CES-D: Center for Epidemiological Studies Depression Scale
CI: confidence interval
IQR: interquartile range
SD: standard deviation
