# Supplementary material for: The Relationship Between Barriers to Physical Activity and Depressive Symptoms in Community-Dwelling Women
Source: Womens Health Rep (New Rochelle). 2024 Mar 13;5(1):242–9. doi: 10.1089/whr.2023.0034 (PMC10956528; doi:10.1089/whr.2023.0034)
Supplement: Supplemental data [file Suppl_Data.docx]

Supplementary material for Figueroa et al.

**Supplementary Figure 1.** The relationship between the Physical Activity Barriers score and depression using a linear spline model


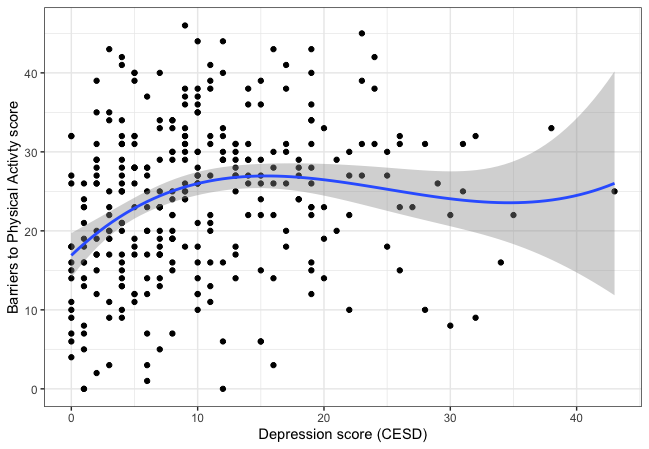


**Supplementary Table 1.** Mean and total barriers subscale scores for women with high and low depression scores (based on CESD cut-off scores of 16). For not normally distributed scales, we present the percentages of women with greater or lower than the sample’s median subscale scores.

|  | **<16 (low depression)** | **≥16 (high depression)** |
| --- | --- | --- |
|  | **(n=245)** | **(n=73)** |
| **Barriers sum score** |  |  |
| Mean (SD) | 23.5 (10.0) | 26.2 (9.65) |
| **Time** |  |  |
| Mean (SD) | 4.12 (2.72) | 4.60 (2.55) |
| **Social Influence** |  |  |
| Mean (SD) | 3.44 (2.09) | 3.82 (2.10) |
| **Lack of energy** |  |  |
| Mean (SD) | 4.04 (2.66) | 4.67 (2.56) |
| **Will power** |  |  |
| <median | 144 (58.8%) | 34 (46.6%) |
| ≥median | 101 (41.2%) | 39 (53.4%) |
| **Fear of injury** | |  |
| <median | 130 (53.1%) | 34 (46.6%) |
| ≥median | 115 (46.9%) | 39 (53.4%) |
| **Lack of skills** | |  |
| <median | 128 (52.2%) | 34 (46.6%) |
| ≥median | 117 (47.8%) | 39 (53.4%) |
| **Lack of resources** | |  |
| <median | 140 (57.1%) | 37 (50.7%) |
| ≥median | 105 (42.9%) | 36 (49.3%) |

Multivariate regression analyses to examine relationships with depression scores and the barrier subscales

**Supplementary Table 2.** Relationship between lack of time and depression scores

|  | **Time** | | |
| --- | --- | --- | --- |
| *Predictors* | *Estimates* | *CI* | *p* |
| (Intercept) | 6.18 | 4.03 – 8.32 | **<0.001** |
| age 10 year^1^ | -0.04 | -0.07 – -0.02 | **0.001** |
| Paid full or part-time employment | 1.30 | 0.65 – 1.95 | **<0.001** |
| Children living at home | 0.16 | -0.55 – 0.86 | 0.661 |
| Never married^2^ | -0.13 | -0.79 – 0.54 | 0.711 |
| Divorced/widowed^2^ | -0.29 | -1.05 – 0.48 | 0.460 |
| Driving in past week | 0.25 | -0.50 – 1.00 | 0.510 |
| BMI | -0.03 | -0.08 – 0.02 | 0.228 |
| Depression scores (CESD) | 0.02 | -0.01 – 0.06 | 0.193 |

**Supplementary Table 3.** Relationship between fear of injury and depression scores

|  | **Fear of Injury** | | |
| --- | --- | --- | --- |
| *Predictors* | *Odds Ratios* | *CI* | *p* |
| (Intercept) | 0.12 | 0.02 – 0.71 | **0.019** |
| age 10 year^1^ | 1.03 | 1.01 – 1.05 | **0.010** |
| Paid full or part-time employment | 1.33 | 0.78 – 2.26 | 0.293 |
| Children living at home | 0.75 | 0.42 – 1.34 | 0.334 |
| Never married^2^ | 0.80 | 0.47 – 1.37 | 0.416 |
| Divorced/widowed^2^ | 1.23 | 0.66 – 2.27 | 0.517 |
| Driving in past week | 0.42 | 0.23 – 0.78 | **0.006** |
| BMI | 1.03 | 0.99 – 1.07 | 0.147 |
| Depression scores (CESD) | 1.02 | 1.00 – 1.05 | 0.100 |

**Supplementary Table 4.** Relationship between lack of skill and depression scores

|  | **Lack of skill** | | |
| --- | --- | --- | --- |
| *Predictors* | *Odds Ratios* | *CI* | *p* |
| (Intercept) | 0.94 | 0.17 – 5.25 | 0.947 |
| age 10 year^1^ | 1.01 | 0.99 – 1.03 | 0.205 |
| Paid full or part-time employment | 0.79 | 0.47 – 1.33 | 0.376 |
| Children living at home | 0.83 | 0.48 – 1.46 | 0.526 |
| Never married^2^ | 0.96 | 0.57 – 1.62 | 0.876 |
| Divorced/widowed^2^ | 0.99 | 0.54 – 1.82 | 0.974 |
| Driving in past week | 0.50 | 0.27 – 0.91 | **0.024** |
| BMI | 0.99 | 0.96 – 1.03 | 0.700 |
| Depression scores (CESD) | 1.03 | 1.00 – 1.06 | 0.057 |

**Supplementary Table 5.** Relationship between lack of resources and depression scores

|  | **Lack of resources** | | |
| --- | --- | --- | --- |
| *Predictors* | *Odds Ratios* | *CI* | *p* |
| (Intercept) | 8.81 | 1.47 – 52.97 | **0.017** |
| age 10 year^1^ | 0.97 | 0.94 – 0.99 | **0.001** |
| Paid full or part-time employment | 1.59 | 0.92 – 2.74 | 0.094 |
| Children living at home | 0.71 | 0.40 – 1.27 | 0.251 |
| Never married^2^ | 1.26 | 0.73 – 2.16 | 0.400 |
| Divorced/widowed_2_ | 0.94 | 0.50 – 1.77 | 0.855 |
| Driving in past week | 0.79 | 0.43 – 1.46 | 0.457 |
| BMI | 0.97 | 0.93 – 1.01 | 0.135 |
| Depression scores (CESD) | 1.03 | 1.00 – 1.06 | 0.061 |

**Supplementary Table 6.** Relationship between lack of willpower and depression scores

|  | **Lack of will power** | | |
| --- | --- | --- | --- |
| *Predictors* | *Odds Ratios* | *CI* | *p* |
| (Intercept) | 3.58 | 0.64 – 20.10 | 0.147 |
| age 10-year^1^ | 0.97 | 0.95 – 0.99 | **0.013** |
| Paid full or part-time employment | 0.86 | 0.51 – 1.45 | 0.570 |
| Children living at home | 0.86 | 0.49 – 1.51 | 0.591 |
| Never married^2^ | 0.92 | 0.54 – 1.56 | 0.755 |
| Divorced/widowed^2^ | 0.68 | 0.36 – 1.26 | 0.220 |
| Driving in past week | 1.15 | 0.63 – 2.10 | 0.658 |
| BMI | 0.99 | 0.96 – 1.03 | 0.735 |
| Depression scores (CESD) | 1.03 | 1.00 – 1.05 | 0.073 |

CES-D: Center for Epidemiological Studies Depression Scale. ^1^Age was divided into 10-year intervals to increase interpretability. ^2^being married was the reference level.
